# Humanness as social normativity: neural evidence that humanized faces align with gender schemas

**DOI:** 10.1093/scan/nsag036

**Published:** 2026-06-12

**Authors:** Fangfang Wen, Yiyan Ju, Yatian Lei, Xierzhati Abulaiti, Min Hu, Bin Zuo

**Affiliations:** School of Psychology, Center for Studies of Social Psychology, Central China Normal University, Wuhan, 430079, China; Key Laboratory of Adolescent Cyberpsychology and Behavior, Ministry of Education, Wuhan, 430079, China; Key Laboratory of Human Development and Mental Health of Hubei Province, Wuhan, 430079, China; School of Psychology, Center for Studies of Social Psychology, Central China Normal University, Wuhan, 430079, China; Key Laboratory of Adolescent Cyberpsychology and Behavior, Ministry of Education, Wuhan, 430079, China; Key Laboratory of Human Development and Mental Health of Hubei Province, Wuhan, 430079, China; School of Psychology, Center for Studies of Social Psychology, Central China Normal University, Wuhan, 430079, China; Key Laboratory of Adolescent Cyberpsychology and Behavior, Ministry of Education, Wuhan, 430079, China; Key Laboratory of Human Development and Mental Health of Hubei Province, Wuhan, 430079, China; School of Psychology, Center for Studies of Social Psychology, Central China Normal University, Wuhan, 430079, China; Key Laboratory of Adolescent Cyberpsychology and Behavior, Ministry of Education, Wuhan, 430079, China; Key Laboratory of Human Development and Mental Health of Hubei Province, Wuhan, 430079, China; School of Psychology, Center for Studies of Social Psychology, Central China Normal University, Wuhan, 430079, China; Key Laboratory of Adolescent Cyberpsychology and Behavior, Ministry of Education, Wuhan, 430079, China; Key Laboratory of Human Development and Mental Health of Hubei Province, Wuhan, 430079, China; School of Psychology, Sun Yat-sen University, Guangzhou, 510006, China

**Keywords:** facial humanness perception, gender schema, event-related potentials (ERP), social norms, mouse-tracking

## Abstract

Perceptions of humanness are deeply intertwined with social norms, yet the specific theoretical link between humanness and gender normativity, a primary form of social normativity, remains insufficiently understood. Across four studies (*N *= 686), we investigated whether humanized faces are implicitly processed as more gender-normative. Study 1 (*n *= 400) revealed that higher humanness ratings predicted stronger femininity evaluations for female faces and stronger masculinity evaluations for male faces. Study 2 (*n *= 161), employing a drawing projective test, demonstrated that highly humanized mental prototypes exhibited more gender-typed traits. In Study 3 (*n *= 92), mouse-tracking revealed that lower Maximum Deviation (MD), an indicator of greater decisional certainty, was linked to evaluations consistent with gender schemas. Study 4 (*n *= 25) provided electrophysiological evidence: in masculinity-evaluation tasks, low-humanness female faces elicited enhanced N170 and LPP amplitudes, indicating increased structural encoding and motivated attention. In femininity-evaluation tasks, low-humanness faces evoked larger P300, suggesting counter-stereotypic processing. Collectively, these findings demonstrate that humanness perception reinforces gender normativity, as reflected in distinct neural signatures.

## Introduction

The human brain is exquisitely tuned for navigating the social world, with face perception serving as a central gateway to interpersonal understanding. Among the many social categories extracted from faces, gender is one of the most salient and automatically processed dimensions, providing a core schema for organizing social reality. Such cognitive structures not only store descriptive knowledge but also carry prescriptive expectations about what it means to be masculine or feminine. While the behavioral outcomes of gender-based perception are well-documented (e.g. Wen and Zuo 2013), less is known about the more fundamental question of when a face is perceived as fully “human,” and how such perceptions shape the application of social norms. Grounded in the perspective that humanness perception is inherently social and normative, we define social normativity as the tendency to evaluate whether a target conforms to socially shared expectations about how individuals should be. We propose that such evaluations depend on perceived humanness, such that entities seen as more human are more likely to be judged according to social normativity. Gender schemas provide a paradigmatic case, as they describe how individuals are expected to appear and behave (e.g. Zuo *et al.* 2021). This research examines whether perceiving a face as more “human” implicitly signals its alignment with gendered social norms, and delineates the temporal dynamics of this process using event-related potentials (ERPs).

Gender schemas are not merely descriptive; they are prescriptive, functioning as potent social norms. The “Big Two” model of social cognition, which decomposes social perception into agency (competence, assertiveness) and communion (warmth, kindness), reveals a deeply gendered underpinning. Agency is associated with masculinity, and communion with femininity (Hsu *et al.* 2021). Recent theoretical advances posit that the “Big Two” dimensions themselves emerge from the fundamental cognitive division of the social world into male and female (Martin and Slepian 2021). This implies that gender schemas serve as a primary lens through which social normative expectations are applied. Consequently, individuals who violate these gendered expectations, such as women displaying dominant traits, often experience social and perceptual penalties, including more negative evaluations (Sutherland *et al.* 2015). The primacy of gender in face perception is well-established: gender is extracted as rapidly as 100–150 ms post-stimulus and automatically gates subsequent social categorical processing (Ito and Urland 2003). This automaticity makes gender schemas an ideal test case for examining the normative dimension of humanness perception.

The attribution of humanness is a fundamental social judgment. Theories of dehumanization posit that fully human characteristics (e.g. secondary emotions and complex language) are denied to out-groups, effectively excluding them from the moral community (Leyens *et al.* 2000). Conversely, the perception of humanness is a defining feature of in-group membership and adherence to social norms (Haslam 2022). Together, these perspectives suggest that humanness perception may function as a gateway for engaging normative social cognition. From a social cognitive neuroscience perspective, the neural circuits processing social norms and person perception are closely intertwined. Neuroimaging studies have consistently implicated a network including the medial prefrontal cortex (mPFC) and temporoparietal junction (TPJ) in mentalizing and normative reasoning. For instance, the mPFC is particularly sensitive to violations of social expectations, a process that is also reflected in electrophysiological measures. Building on these findings, we hypothesize that perceiving a face as highly “human” engages neural systems associated with normative cognition. This, in turn, facilitates evaluations that are consistent with gender schemas.

Building on this foundation, the present research advances a theoretical framework we term “*humanness as social normativity*.” This framework posits that the attribution of humanness operates as a higher-order cognitive signal indicating when a target is subject to fundamental social expectations. Importantly, rather than assessing how well an individual matches specific category-based stereotypes, humanness functions as a core criterion governing whether and how strongly normative expectations are applied. Under this framework, gender schemas serve as a central organizing principle of social normativity: because gender is one of the most automatically activated social categories, the perception of humanness should be systematically associated with gender-typical attributes. Critically, this framework generates several distinct predictions: (a) faces perceived as more human will be evaluated as more gender-normative; (b) violations of this humanness-gender link will elicit cognitive conflict; and (c) this norm-based process will manifest across multiple levels of analysis, from mental representations to neuro-temporal dynamics.

Within this framework, gender schemas provide a particularly suitable test case. Gender represents a fundamental organizing principle of social cognition, serving as a primary lens through which individuals are categorized and evaluated (Martin and Slepian 2021). Recent theoretical work by Martin and Mason (2022) further suggests that gender is not only rapidly extracted but also prioritized in humanness perception, exhibiting a perceptual advantage over other social categories. Moreover, gender norms are uniquely prescriptive: they specify not only how individuals are likely to behave (descriptive norms) but also how they ought to behave (prescriptive norms; Prentice and Carranza 2002). This prescriptive quality makes gender schemas a paradigmatic case of social normativity. By demonstrating that humanness perception aligns with gender expectations, we establish a foundation for understanding how humanness operates as a broader normative mechanism, one that may extend to other socially meaningful and normatively regulated categories, such as race, age, and social class.

Emerging evidence specifically links humanness perception to gendered expectations. Recent work has demonstrated that gender is a central and prioritized feature in humanness perception, showing a perceptual advantage over other social categories (Martin and Mason 2022). Furthermore, violating gender norms can lead to a form of perceptual dehumanization; faces that counter stereotypes are not only evaluated more negatively but are also processed as less normative (Fincher and Tetlock 2016). Recent ERP evidence further shows that when moral beauty and facial beauty conflict, moral beauty dominates both social judgments and emotional brain responses (Baum & Abdel Rahman, 2024). This resistance to counter-stereotypes suggests that humanness perception and gender schema conformity may rely on shared neurocognitive pathways. However, the electrophysiological correlates of this relationship, which are critical for understanding the rapid, cascading processes of social perception, remain largely unexplored.

The high temporal resolution of ERPs makes them particularly well-suited for examining the rapid dynamics of face perception and social evaluation (Hajcak *et al.* 2019). Three components are especially relevant for testing the “humanness as social normativity” hypothesis.

The N170, a negative peak occurring around 170 ms over temporo-occipital sites, is a robust marker of the structural faces encoding (Eimer 2000). While primarily sensitive to face configuration, its modulation can also reflect the extraction of socially salient category information, including gender atypicality (Freeman *et al.* 2010). We hypothesize that faces violating gender-based humanness expectations will require more complex structural processing, manifesting as enhanced N170 amplitudes.

The P300 is a positive deflection peaking around 300–400 ms over parietal regions that has been linked to context updating and the categorization of motivationally relevant stimuli (Polich 2012). Critically, P300 amplitudes are sensitive to stereotype violations, with counter-stereotypic information consistently eliciting larger responses than stereotypic information, a pattern documented in the context of gender stereotypes (Serafini and Pesciarelli 2026). In the present study, low-humanness faces may be perceived as counter-stereotypic, thereby evoking enhanced P300 amplitudes.

The Late Positive Potential (LPP), a sustained positivity from ∼500 to 700 ms over central-parietal sites, reflects motivated attention and the enhanced processing of emotionally and motivationally significant stimuli (Hajcak and Foti 2020). LPP amplitudes are typically increased when stimuli violate expectations or demand deeper evaluative processing. We predict that faces low in humanness, as potential violations of gender norms, will elicit larger LPP amplitudes, indicating heightened attentional engagement.

Together, these components provide a fine-grained temporal perspective on how humanness may influence gender evaluation: from early structural analysis (N170), through higher-level cognitive appraisal of stereotype consistency (P300), to motivated attentional engagement (LPP).

The present research employs a four-study, multi-method design to test the humanness-as-social-normativity framework. Study 1 establishes the basic behavioral relationship between face humanness and gender-typed evaluations using explicit ratings. Study 2 extends this investigation to implicit mental representations using a projective drawing task, testing whether highly humanized mental prototypes spontaneously exhibit gender-typical features. Study 3 employs mouse-tracking to capture the real-time cognitive dynamics of humanness judgments, examining whether gender-atypical features interfere with fluent humanness categorization as reflected in trajectory dynamics. Finally, Study 4 utilizes event-related potentials (ERPs) to delineate the neuro-temporal signatures of this process, from early structural encoding (N170) through motivated attention (LPP) to stereotype-violation processing (P300). This progression, from explicit behavioral measures, to implicit representations, to dynamic cognitive processing, and finally to neural temporal dynamics, provides convergent, multi-level evidence for the proposed role of humanness as a mechanism underlying gender normativity.

By linking the social psychology of humanness and gender norms with the precise temporal metrics of brain activity, this research aims to provide a novel neurocognitive account of “humanness as social normativity,” illuminating the brain’s implicit reliance on social norms when perceiving the humanity of others.

## Study 1: The relationship between facial humanness perception and sexual dimorphism evaluations

### Purpose

Study 1 employed a face trait evaluation paradigm to investigate whether the perception of humanness in neutral faces predicts their subsequent evaluations of masculinity and femininity. We hypothesized that higher perceptions of humanness would be associated with stronger masculinity evaluations for male faces and stronger femininity evaluations for female faces, indicating a conformity to gender schemas.

### Participants

We recruited 400 participants (200 females; *M*_age_ = 21.32, *SD *= 2.96) via an online survey platform (Credamo). All participants reported no history of mental illness and had normal or corrected-to-normal vision. A priori power analysis was conducted using the *pwr* package in RStudio (Champely 2020). With a medium effect size (*f*^2^ = 0.15), a statistical power of 0.8, and a significance level of *α* = .05, the analysis indicated a minimum requirement of 76 facial stimuli. The choice of medium effect size was guided by prior research on face perception and social categorization, which typically yields medium-sized effects for gender-based evaluations (Freeman *et al.* 2010, Sutherland *et al.* 2015). Our study utilized 80 faces, exceeding this threshold.

### Materials

Facial stimuli were selected from the Chinese Affective Face Picture System (CAFPS). We chose 40 male and 40 female faces with neutral expressions, based on the descending order of emotional recognition accuracy within the database. All images were standardized to a size of 340 × 512 pixels. For the online presentation, images were scaled to 40% of their original size on the questionnaire interface.

### Procedure

After providing informed consent, participants first completed a demographic survey (age, gender). Subsequently, they were presented with five randomly selected faces of the same gender (from the total set of 80) and rated each on three seven-point scales (1 = very disagreeable, 7 = very agreeable) for humanness, masculinity, and femininity. Each face was rated by at least 25 participants.

**Figure 1:**
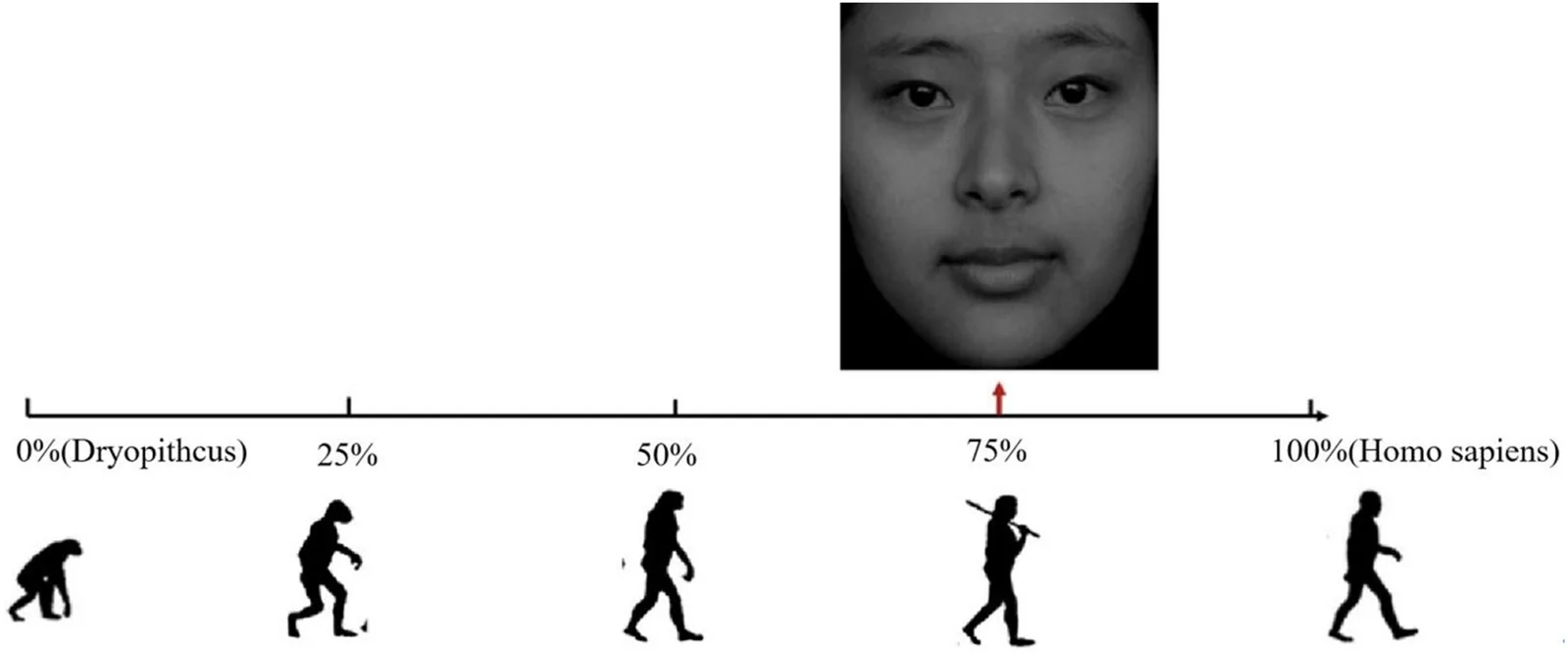
The Ascent measure of blatant dehumanization. Responses were made for each target face. Target face order was randomized across participants. Adapted from (Kteily et al. 2015)

The perception of humanness was measured using an adapted version of the Overt Dehumanization Scale (Fig. 1). Participants were instructed: “People vary in the degree to which they look human. Some people look highly evolved, while others look indistinguishable from lower animals. We ask you to rate how evolved the people in the picture are. There is no right or wrong answer; rely on your intuition (drag the slider to a position that matches what you really think).” Responses were recorded on a slider ranging from 0% (labeled with an image of Dryopithecus, a stooped ancestral ape) to 100% (labeled with an image of an upright walking Homo sapiens).

### Results

Data analysis was performed in RStudio (*lme4* package), with evaluations of masculinity and femininity as dependent variables. Participants and faces were included as random effects in the models. The descriptive statistics for all evaluations are summarized in Table 1.

**Table 1:** Descriptive statistics (M ± SD) for target face ratings in Study 1.

Evaluation indicators	Face gender	Participant gender	
		Female	Male
Masculinity	Female	3.55 ± 1.51	3.78 ± 1.56
	Male	5.49 ± 1.36	5.36 ± 1.38
Femininity	Female	4.87 ± 1.35	4.42 ± 1.55
	Male	2.48 ± 1.43	2.54 ± 1.44

#### Masculinity evaluation

A linear mixed model revealed a significant negative main effect of humanness perception on masculinity evaluation, *b *= −0.02, *SE *= 0.004, *t*(1725.91) = −6.68, *P* < .001. As predicted, significant interactions were observed between humanness perception and face gender, *b *= 0.04, *SE *= 0.01, *t*(1736.77) = 7.49, *P* < .001, and between humanness perception and participant gender, *b *= 0.02, *SE *= 0.005, *t*(1658.64) = 3.21, *P *= 0.001. The three-way interaction among humanness perception, face gender, and participant gender was also significant, *b *= −0.01, *SE *= 0.007, *t*(1641.89) = −2.02, *P* = .044.

Simple slope analyses were conducted to decompose these interactions. For female participants, humanness perception negatively predicted masculinity evaluation for female faces, *b *= −0.02, *SE *= 0.001, *t *= −6.68, *P* < .001, but positively predicted masculinity evaluation for male faces, *b *= 0.01, *SE *= 0.001, *t *= 3.79, *P* < .001. A similar pattern was found for male participants: humanness perception negatively predicted masculinity evaluation for female faces, *b *= −0.01, *SE *= 0.001, *t *= −2.89, *P* < .001, and positively predicted it for male faces, *b *= 0.01, *SE *= 0.001, *t *= 4.33, *P* < .001.

##### Femininity evaluation

For femininity evaluation, the model showed a significant positive main effect of humanness perception, *b *= 0.03, *SE *= 0.004, *t*(1938.61) = 9.08, *P* < .001. A significant interaction emerged between humanness perception and face gender, *b *= −0.03, *SE *= 0.005, *t*(1949.18) = −7.04, *P* < .001. The three-way interaction among humanness perception, face gender, and participant gender was also significant, *b *= 0.01, *SE *= 0.01, *t*(1850.44) = 1.97, *P* = .049.

Simple slope analyses revealed that for both female and male participants, humanness perception significantly and positively predicted femininity evaluation for female faces (Female participants: *b *= 0.03, *SE *= 0.001, *t *= 9.08, *P* < .001; Male participants: *b *= 0.02, *SE *= 0.001, *t *= 7.60, *P* < .001). However, humanness perception did not significantly predict femininity evaluation for male faces, regardless of the participant’s gender (*p*s = .492 to .687).

### Discussion

The findings from Study 1 provide initial behavioral evidence that the perception of humanness in faces is systematically linked to gender-based trait evaluations. As the perceived humanness of a face increased, so did its alignment with gender schemas: male faces were evaluated as more masculine, and female faces were evaluated as more feminine. Conversely, higher humanness was associated with lower masculinity evaluations for female faces. This pattern suggests that humanness perception may act as a higher-order social cognitive cue, implicitly signaling adherence to fundamental social norms, including those governing gender.

This study establishes a crucial behavioral foundation for the subsequent investigations in our research series, which will employ more implicit measures (projective drawing, mouse-tracking) and electrophysiological methods (ERP) to further delineate the cognitive and neural mechanisms underlying this phenomenon.

## Study 2: Mental prototypes of highly humanized faces: evidence from a face projective test

### Purpose

Study 1 established a correlational link between humanness perception and gender-typed evaluations using explicit rating scales. To probe the mental representations of highly humanized faces more implicitly, Study 2 employed a face projective test in the form of a free-drawing task. We hypothesized that when individuals visualize and draw a “highly humanized” face, their mental prototypes would spontaneously exhibit traits congruent with gender schemas: highly humanized male drawings would be rated as more masculine, and highly humanized female drawings would be rated as more feminine.

### Participants

A total of 161 participants (99 females; *M*_age_ = 19.31, *SD *= 0.81) were recruited for this offline study. A power analysis was conducted using the *pwr* package in RStudio. With a preset medium effect size (*f^2^* = 0.15), a statistical power of 1−*β* = 0.8, and *α* = 0.05, the analysis indicated a minimum of 45 participants per group. Our final sample of 161 provided adequate power for the planned analyses.

### Study design

The study employed a 2 (Participant Gender: male, female) × 2 (Drawn Face Gender: male, female) between-subjects design. The dependent variables were the ratings of masculinity and femininity of the drawn faces.

### Materials and procedure

The study utilized blank A4 paper and a neutral-colored pen. The instruction for the drawing task was: “Please use the pen to draw a highly humanized face image in your mind on this blank A4 paper. Any character is okay. Please try your best to draw until you are satisfied.”

**Figure 2:**
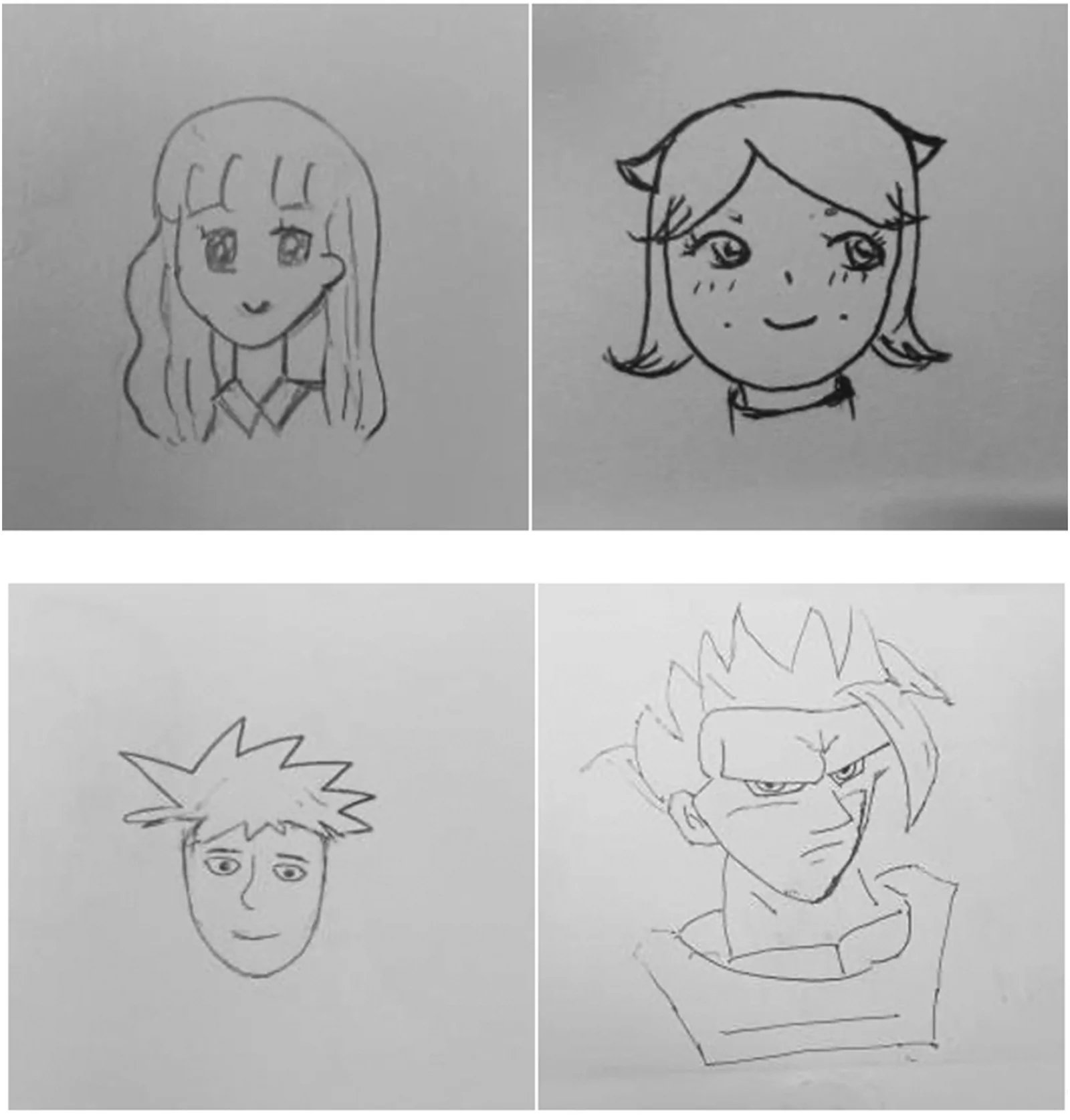
Example drawings from the projective drawing task in Study 2. Participants created face drawings based on the experimental instructions. The figure presents representative examples reflecting variations in perceived humanness and gender-related features. These drawings are shown for illustration only and are not exhaustive of all responses.

After completing their drawings, participants were asked to rate the masculinity, femininity, and humanness of the figures on seven-point scales (1 = Very Low, 7 = Very High). This allowed us to verify that the drawn faces were indeed perceived as highly humanized and to collect the key dependent measures. Example drawings from participants are presented in Fig. 2.

### Results

Data were analyzed using the MANOVA function of the *bruceR* package (Bao 2023) in RStudio. The descriptive statistics for the masculinity and femininity ratings of the drawn faces are summarized in Table 2.

**Table 2:** Descriptive statistics (M ± SD) for ratings of drawn faces in study 2.

Evaluation indicators	Draw face gender	Participant gender	
		Female	Male
Masculinity	Female	2.58 ± 1.29	2.60 ± 1.59
	Male	5.27 ± 1.56	5.50 ± 2.12
Femininity	Female	5.72 ± 1.18	5.80 ± 0.84
	Male	2.38 ± 1.52	2.49 ± 1.82

### Masculinity evaluation

A two-way ANOVA with masculinity evaluation as the dependent variable revealed a significant main effect of drawn face gender, *F*(1, 155) = 61.80, *P* < .001, *η_p_*^2^ = .29. As predicted, highly humanized male drawings (*M *= 5.54, *SE *= 0.16) received significantly higher masculinity ratings than highly humanized female drawings (*M *= 2.59, *SE *= 0.34). The main effect of participant gender and the interaction was not significant (*p*s = .812 to .894).

### Femininity evaluation

Similarly, for femininity evaluation, the ANOVA showed a significant main effect of drawn face gender, *F*(1, 155) = 74.98, *p* < .001, *η_p_*^2^ = .33. Highly humanized female drawings (*M *= 5.76, *SE *= 0.35) were rated as significantly more feminine than highly humanized male drawings (*M *= 2.44, *SE *= 0.16). Neither the main effect of participant gender nor the interaction was significant (*p*s = .803 to .856).

### Discussion

Study 2 leveraged the projective drawing task to access mental prototypes of highly humanized faces. The results were clear and consistent: the highly humanized faces spontaneously generated by participants’ minds were strongly gendered. Male drawings were ascribed higher masculinity, and female drawings were ascribed higher femininity. This finding conceptually replicates and extends the results of Study 1 by moving beyond explicit ratings of static faces to a more implicit measure of internal representation.

The act of drawing a “highly humanized” face appears to automatically engage and externalize gender schemas. This provides compelling evidence that the link between humanness perception and gender norms is not merely a product of explicit rating biases. Instead, it is deeply embedded in our cognitive architecture, influencing even the content of our mental imagery. This study sets the stage for the subsequent investigation in Study 3, which employs another implicit measure, mouse-tracking, to capture the dynamic cognitive processes during humanness judgments.

One potential limitation of the drawing paradigm warrants consideration. The explicit instruction to draw a “highly humanized” face may raise concerns about potential demand characteristics, prompting participants to generate culturally prototypical gendered features. However, two aspects of the data mitigate this concern. First, the spontaneous nature of free drawing, participants received no guidance on specific features to include, suggests that the gendered content reflects automatic activation of mental prototypes rather than deliberate conformity to task demands. Second, the convergence between implicit projective measure in Study 2 and explicit ratings in Study 1, as well as the implicit mouse-tracking dynamics in Study 3, supports the interpretation that the humanness-gender link is robust across measurement contexts and not merely driven by task-specific demand characteristics. Nevertheless, future research could employ more indirect priming paradigms to further minimize potential demand characteristics.

## Study 3: Implicit cognitive dynamics of humanness judgments and gender schema: a mouse-tracking investigation

### Purpose

Building on the explicit and projective evidence from Studies 1 and 2, Study 3 employed mouse-tracking, a dynamic behavioral method sensitive to real-time cognitive competition, to further examine the association between humanness perception and gender schema. We hypothesized that evaluating faces violating gender-based humanness expectations (e.g. low-humanness male faces or high-humanness female faces in masculinity judgments) would induce greater response conflict, manifesting as increased curvature in mouse trajectories toward the unselected alternative.

### Participants

Ninety-two participants (50 females; *M*_age_ = 19.78, *SD *= 1.50) were recruited. All reported no history of neurological or psychiatric conditions and had normal or corrected-to-normal vision. A sensitivity analysis conducted using the *pwr* package in R indicated that this sample size provided 84% power to detect a medium-sized effect (*f^2^* = 0.15) at *α*  =  0.05.

### Materials and procedure

Stimuli consisted of 60 faces (15 high-humanness male, 15 low-humanness male, 15 high-humanness female, 15 low-humanness female) selected from the CAFPS based on normative ratings collected in a pilot study. Humanness categorization was performed using MouseTracker software. On each trial, participants initiated from a “Start” button at the bottom center of the screen, after which a face stimulus appeared centrally. They were instructed to categorize the face as “Humanized” or “Dehumanized” by clicking the corresponding button in the top-left or top-right corner, with button position counterbalanced across participants.

Mouse trajectories were recorded at 70 Hz. Maximum deviation (MD), quantified as the largest perpendicular distance (in normalized MouseTracker units) between the actual trajectory and the idealized direct path to the selected endpoint, served as the primary index of cognitive conflict. Higher MD values reflect greater attraction toward the unselected category, indicating simultaneous activation of competing responses.

Following the mouse-tracking task, participants provided explicit masculinity and femininity ratings for all faces using E-Prime 2.0, with rating order randomized across participants.

### Results

#### Manipulation check

A chi-square test confirmed successful experimental manipulation, *χ*^2^(1) = 84.77, *P* < .001, indicating that participants’ humanness judgments aligned with the a priori classification of faces.

#### Mouse-tracking analysis

Linear mixed-effects models were fitted with masculinity/femininity ratings as dependent variables, and MD, face gender, participant gender, and their interactions as fixed effects, including random intercepts for participants and faces.

For masculinity evaluation, a significant MD × face gender interaction emerged, *b *= −0.30, *SE *= 0.15, *t*(2651.04) = −2.04, *P* = .042. Simple slopes analysis revealed that for male faces, MD negatively predicted masculinity ratings, *b *= −0.20, *SE *= 0.11, *t *= −1.81, *P* = .070, whereas for female faces, MD showed no significant relationship, *b *= 0.10, *SE *= 0.11, *t *= 0.99, *P* = .320.

For femininity evaluation, a significant MD × face gender interaction was also observed, *b *= 0.34, *SE *= 0.12, *t*(2623.45) = 2.81, *P* = .005. Here, MD negatively predicted femininity ratings for female faces, *b *= −0.24, *SE *= 0.09, *t *= −2.79, *P* = .010, but not for male faces, *b *= 0.10, *SE *= 0.09, *t *= 1.10, *P* = .270.

### Discussion

Study 3 provides implicit behavioral evidence for the dynamic conflict underlying humanness and gender schema evaluations. The mouse-trajectory findings demonstrate that lower masculinity in male faces and lower femininity in female faces, both representing deviations from gender-typed humanness, elicit greater cognitive competition during humanness judgments, as reflected in increased MD. This pattern suggests that gender-atypical features interfere with the fluid categorization of a face as “humanized,” implicitly reinforcing the normative link between humanness and gender schema consistency. These results complement the explicit and projective measures of Studies 1–2 and set the stage for examining the neural temporal dynamics of these processes in Study 4.

## Study 4: Neural dynamics of gender schema processing in humanness perception: an ERP investigation

### Purpose

Employing event-related potentials (ERPs), Study 4 sought to identify the neuro-temporal signatures underlying the evaluation of gender-typed traits in faces varying in perceived humanness. We specifically examined three components: N170 (structural encoding), LPP (motivated attention), and P300 (stereotype violation processing), to test the hypothesis that low-humanness faces violating gender norms would elicit enhanced neural responses associated with complex social-cognitive evaluation.

### Participants

Twenty-five participants (11 females; *M*_age_ = 21.05, *SD *= 2.59) met all inclusion criteria following data quality screening. Sample size was determined based on prior ERP studies examining face perception and social categorization, which typically employ sample sizes of 20–25 participants to detect medium-to-large effects (Carrito *et al.* 2018, Baum and Rahman 2024). A sensitivity analysis using G*Power 3.1 indicated that with *n *= 25, *α* = .05, and correlation among repeated measures *r* = .50, we had 80% power to detect a medium-sized effect (*f *= 0.25) for the critical interactions.

### Stimuli and procedure

The face stimulus set was identical to Study 3. Participants completed both masculinity and femininity evaluation tasks while EEG was recorded. Each trial began with a central fixation cross (250–550 ms), followed by a face stimulus (600 ms). Participants then provided gender-trait ratings using a 7-point scale (1 = Very Disagree, 7 = Very Agree), with no time limit. Stimuli were presented in blocks by humanness level and gender, with each block repeated three times. Trial order was randomized within blocks, and rating dimension order was counterbalanced across participants.

### EEG recording and preprocessing

Continuous EEG was recorded from 64 Ag/AgCl electrodes positioned according to the 10-20 system, referenced to FCz with a ground at AFz. The vertical electrooculogram was recorded from an electrode below the right eye. Data were sampled at 500 Hz with a bandpass filter of 0.1–100 Hz. Offline processing included re-referencing to average mastoids, bandpass filtering (0.1–30 Hz), and ocular artifact correction using ICA. Epochs were extracted from −200 to 800 ms relative to stimulus onset, baseline-corrected (−200 to 0 ms), and automatically rejected for artifacts exceeding ±75 μV.

### ERP quantification

Based on previous literature and visual inspection of scalp topographies, we analyzed: N170 (mean amplitude 170–220 ms at P7, P8); LPP (mean amplitude 500–700 ms at CP1, CP2, Pz); and P300 (mean amplitude 240–380 ms at CP1, CP2, CPz, P1, P2, Pz). Our choice of the central-parietal electrode cluster for P300 quantification follows established practice in social neuroscience. This component reaches its maximal amplitude over midline and adjacent centro-parietal sites (Hajcak and Foti 2020), and the particular combination of CP1, CP2, CPz, P1, P2, and Pz has been routinely adopted in studies of social categorization and face processing (Serafini and Pesciarelli 2025).

### Results

#### Operational check

An independent samples t-test confirmed that high-humanness faces (*M *= 5.43, *SD *= 1.43) received significantly higher humanness ratings than low-humanness faces (*M *= 4.82, *SD *= 1.61), *t*(2815) = −10.66, *P* < .001, Cohen’s *d *= −0.40.

### Evaluation task

#### N170 (170–220 ms)

N170 analysis revealed a significant three-way interaction between participant gender, face gender, and humanness, *F*(1,22) = 4.47, *P* = .046, *η_p_*^2^ = 0.17. For female participants, low-humanness male faces elicited significantly larger N170 amplitudes than high-humanness male faces (*P* = .036).

#### P300 (240–380 ms)

In the femininity evaluation task, P300 analysis revealed a significant main effect of humanness, *F*(1,22) = 5.94, *P* = .023, *η_p_*^2^ = 0.21, with low-humanness faces eliciting larger amplitudes than high-humanness faces. No significant P300 effects emerged in the masculinity evaluation task.

#### LPP (500–700 ms)

In the masculinity evaluation task, LPP analysis showed a significant face gender × humanness interaction, *F*(1,22) = 4.62, *P* = .043, *η_p_*^2^ = 0.17. Low-humanness female faces elicited larger LPP amplitudes than high-humanness female faces (*P* = .017). This was qualified by a three-way interaction, *F*(1,22) = 4.90, *P* = .038, *η_p_*^2^ = 0.18, indicating the effect was primarily driven by female participants. No significant LPP effects were observed in the femininity evaluation task.

### Discussion

Study 4 provides crucial electrophysiological evidence for the neural dynamics underlying gender schema processing in humanness perception. Three key findings emerge:

First, the enhanced N170 for low-humanness male faces during masculinity evaluation suggests increased computational demands in structural encoding when male faces lack typical masculine features. This aligns with models proposing that N170 reflects configural processing of socially relevant facial information.

Second, the amplified LPP for low-humanness female faces during masculinity evaluation indicates heightened motivated attention toward faces that doubly violate expectations, both in terms of humanness and gender norms. This pattern is consistent with literature linking LPP enhancement to motivational significance and affective engagement.

Third, the generalized P300 enhancement for low-humanness faces during femininity evaluation suggests a domain-general sensitivity to norm violations, possibly reflecting the updating of mental models when faces deviate from expected gender-humanness associations.

These neural signatures offer a temporally precise account of how gender schemas shape humanness perception across multiple processing stages, from early structural encoding to late evaluative categorization. They complement and extend the behavioral results from Studies 1-3 by revealing the distinct neuro-temporal dynamics that underlie the implicit association between humanness perception and gender normativity.

The gender-asymmetric ERP effects observed in Study 4 merit further discussion. Enhanced N170 and LPP for low-humanness female faces during masculinity evaluation, effects driven primarily by female participants, suggest potential ingroup sensitivity to gender norm violations. This pattern aligns with research on social identity and neural processing, which demonstrates that individuals are particularly attuned to expectancy violations involving ingroup members (Molenberghs and Morrison 2014). Female participants may exhibit heightened motivated attention (reflected in LPP) when evaluating female targets who deviate from masculine expectations because such violations have greater self-relevance. Alternatively, this asymmetry may also reflect gender differences in the prescriptive rigidity of social norms, whereby masculine norms may be enforced more strictly for men, potentially leading to different neural dynamics. Future research should systematically manipulate both perceiver gender and target gender to disentangle ingroup sensitivity effects from domain-general norm violation detection.

The distinct ERP patterns observed across masculinity-evaluation and femininity-evaluation tasks also warrant theoretical consideration. In masculinity-evaluation tasks, low-humanness female faces elicited enhanced N170 and LPP amplitudes, suggesting that evaluating female faces for masculine traits, a dimension incongruent with the target’s gender category, engages both early structural encoding and later motivated attention. In contrast, femininity-evaluation tasks showed generalized P300 enhancement for low-humanness faces regardless of target gender, indicating a broader sensitivity to expectancy violations when the evaluated dimension aligns with the female gender category. This dissociation may reflect asymmetric processing demands, such that evaluating counter-stereotypic traits (masculinity in female faces) may require sustained attentional engagement (LPP), whereas evaluating stereotypic traits (femininity) may involve rapid context updating (P300) when expectations are violated. These findings illustrate how humanness-based normativity operates differently depending on target gender and evaluative context.

## General discussion

Across four multi-method studies, our findings consistently demonstrate that faces perceived as more human are implicitly processed as more gender-normative, while deviations from this pattern elicit distinct behavioral conflicts and neuro-temporal signatures. By positioning humanness within the framework of social normativity, this work reveals that humanness perception is not merely a descriptive judgment, but a process that regulates how social expectations are applied across multiple levels of analysis. Importantly, this perspective distinguishes humanness from mere stereotype conformity. Rather than reflecting the extent to which a target matches category-based expectations, humanness serves as a higher-order cue that determines whether and how strongly such expectations are applied. Thus, to perceive a target as fully human is, in part, to hold it accountable to the normative standards that structure social cognition.

The convergence of findings across Studies 1–3 provides robust evidence that the humanness–gender schema link operates across multiple levels of processing. At the explicit level, humanness perception systematically predicts gender-typed evaluations, with higher humanness amplifying perceived masculinity in male faces and femininity in female faces (Study 1). At the representational level, projective drawings demonstrated that this association is deeply embedded in mental representations (Study 2): when individuals visualize “highly humanized” faces, they spontaneously generate gender-prototypical features (Martin and Slepian 2021). At the implicit level, mouse-tracking captured the dynamic cognitive conflict underlying these judgments, showing that gender-atypical features interfere with the fluid categorization of faces as humanized (Study 3). Together, these studies suggest that the humanness-gender schema link operates across explicit, representational, and implicit processing levels.

The ERP findings from Study 4 provide crucial neuro-temporal precision to this framework, revealing distinct neural dynamics across processing stages. Early structural encoding (N170) was sensitive to violations of gender-based humanness expectations, suggesting increased perceptual demands when faces deviate from normative configurations. Later components associated with motivated attention (LPP) and context updating (P300) were also modulated by these violations, indicating that norm-based processing extends beyond early perception to sustained evaluative stages. Taken together, these neural patterns suggest that the application of social norms in humanness perception is temporally distributed, spanning from initial perceptual encoding to later attentional and evaluative processes.

### Theoretical implications

From a theoretical perspective, the present findings advance existing accounts of humanness and social cognition in several important ways. First, the article broadens dehumanization theory by demonstrating that humanness perception operates not merely as an intergroup phenomenon but as a fundamental normative process guiding basic face perception (Haslam 2022). Even in the absence of explicit group boundaries, faces are evaluated against implicit standards of what it means to appear “fully human,” standards that are closely tied to gendered expectations.

Second, these findings contribute to emerging research on the neural basis of norm enforcement. Recent work by Serafini and Pesciarelli (2025) demonstrated that gender-stereotypical primes influence face processing at multiple ERP stages, including P300 and LPP. Our results complement and extend these findings by showing that humanness itself functions as a normative signal, with low-humanness faces eliciting neural signatures similar to those observed for stereotype-incongruent information. This suggests that the brain may employ common computational mechanisms for detecting violations of both explicit stereotypes and more implicit normativity signals.

Third, this research extends the “Big Two” model of social cognition by suggesting that agency and communion may be downstream consequences of more fundamental humanness-based normative processing (Hsu *et al.* 2021). Our work suggests that to be perceived as fully human is, in part, to conform to the gendered expectations that structure social perception.

### Limitations and future directions

Several limitations of the present research should be noted. First, our samples consisted of Chinese young adults, a population situated within a cultural context characterized by relatively traditional gender role expectations (Blair and Madigan 2016). Research on cultural tightness-looseness suggests that societies with stronger norms exhibit tighter coupling between category-based expectations and social perception (Gelfand *et al.* 2011). Future cross-cultural research should examine whether the humanness-gender schema link generalizes to more gender-egalitarian cultural contexts, or whether the neural signatures observed here, particularly the enhanced N170 and LPP for norm violations, are attenuated in cultures with looser gender expectations.

Second, although the multi-method design reduces the likelihood that the findings are driven by task-specific artifacts, certain paradigms (e.g. projective drawing) may still introduce potential demand characteristics. While converging evidence across methods mitigates this concern, future work using more indirect paradigms would further strengthen causal inference.

Notably, the gender asymmetry in some neural effects, particularly the distinct patterns for male and female faces across different ERP components, suggests complex interactions between target gender and evaluation context. This specificity underscores the importance of moving beyond broad humanness judgments to examine how social category information modulates neuro-cognitive processing across different evaluative contexts.

Several specific future directions emerge from this work. First, combining ERP with fMRI could localize the neural sources of these effects, particularly in regions implicated in normative reasoning such as medial prefrontal cortex and temporoparietal junction. Second, developmental studies could examine when the humanness-gender link emerges and how it is shaped by socialization. Third, interventions that disrupt automatic gender stereotyping could be tested for their effects on humanness perception, potentially revealing causal relationships.

In conclusion, this research provides a multi-level account of how humanness perception reinforces social norms through gender schema consistency. By integrating behavioral, implicit, projective, and neuro-temporal methods, we demonstrate that the perception of humanness is intrinsically normative, what we see as “human” is deeply shaped by what we expect from gendered social beings. In essence, to perceive a target as human is not merely to recognize it as a person, but to subject it to the normative expectations that define social life. These findings open new avenues for understanding how fundamental social categories become woven into the fabric of human perception, with implications for research on social cognition, stereotyping, and the neural basis of norm enforcement.

## Data Availability

The data that support the findings of this study are available from the corresponding author upon reasonable request.
